# Metal-Based Nanoparticles as Antimicrobial Agents: An Overview

**DOI:** 10.3390/nano10020292

**Published:** 2020-02-09

**Authors:** Elena Sánchez-López, Daniela Gomes, Gerard Esteruelas, Lorena Bonilla, Ana Laura Lopez-Machado, Ruth Galindo, Amanda Cano, Marta Espina, Miren Ettcheto, Antoni Camins, Amélia M. Silva, Alessandra Durazzo, Antonello Santini, Maria L. Garcia, Eliana B. Souto

**Affiliations:** 1 Department of Pharmacy, Pharmaceutical Technology and Physical Chemistry, Faculty of Pharmacy, University of Barcelona, 08028 Barcelona, Spain; gerard.esteruelas.n@gmail.com (G.E.); bonilla.vidal95@gmail.com (L.B.); lora_ana@hotmail.com (A.L.L.-M.); ruth.galindo@ub.edu (R.G.); amanda90cf@gmail.com (A.C.); m.espina@ub.edu (M.E.); marisagarcia@ub.edu (M.L.G.); 2 Institute of Nanoscience and Nanotechnology (IN2UB), University of Barcelona, 08028 Barcelona, Spain; 3 Networking Research Centre of Neurodegenerative Disease (CIBERNED), Instituto de Salud Juan Carlos III, 28031 Madrid, Spain; e_miren60@hotmail.com (M.E.); camins@ub.edu (A.C.); 4 Faculty of Pharmacy (FFUC), Department of Pharmaceutical Technology, University of Coimbra, Pólo das Ciências da Saúde, Azinhaga de Santa Comba, 3000-548 Coimbra, Portugal; dlsgomes@live.com.pt; 5 Department of Pharmacology and Therapeutic Chemistry, Faculty of Pharmacy, University of Barcelona, 08028 Barcelona, Spain; 6 Department of Biology and Environment, University of Trás-os-Montes e Alto Douro, UTAD, Quinta de Prados, P-5001-801 Vila Real, Portugal; amsilva@utad.pt; 7 Centre for Research and Technology of Agro-Environmental and Biological Sciences, CITAB, UTAD, Quinta de Prados, P-5001-801 Vila Real, Portugal; 8 CREA—Research Centre for Food and Nutrition, Via Ardeatina 546, 00178 Rome, Italy; alessandra.durazzo@crea.gov.it; 9 Department of Pharmacy, University of Napoli Federico II, Via D. Montesano 49, 80131 Napoli, Italy; asantini@unina.it; 10 CEB—Centre of Biological Engineering, University of Minho, Campus de Gualtar, 4710-057 Braga, Portugal

**Keywords:** antibacterial activity, metal-based nanoparticles, AgNPs, CuONPs, AuNPs, ZnONPs

## Abstract

Metal-based nanoparticles have been extensively investigated for a set of biomedical applications. According to the World Health Organization, in addition to their reduced size and selectivity for bacteria, metal-based nanoparticles have also proved to be effective against pathogens listed as a priority. Metal-based nanoparticles are known to have non-specific bacterial toxicity mechanisms (they do not bind to a specific receptor in the bacterial cell) which not only makes the development of resistance by bacteria difficult, but also broadens the spectrum of antibacterial activity. As a result, a large majority of metal-based nanoparticles efficacy studies performed so far have shown promising results in both Gram-positive and Gram-negative bacteria. The aim of this review has been a comprehensive discussion of the state of the art on the use of the most relevant types of metal nanoparticles employed as antimicrobial agents. A special emphasis to silver nanoparticles is given, while others (e.g., gold, zinc oxide, copper, and copper oxide nanoparticles) commonly used in antibiotherapy are also reviewed. The novelty of this review relies on the comparative discussion of the different types of metal nanoparticles, their production methods, physicochemical characterization, and pharmacokinetics together with the toxicological risk encountered with the use of different types of nanoparticles as antimicrobial agents. Their added-value in the development of alternative, more effective antibiotics against multi-resistant Gram-negative bacteria has been highlighted.

## 1. Introduction

Bacteria were the first living organisms found on the Earth and they have become highly adaptable over the course of time. During the 20th century, the discovery of antibiotics was considered one of the most significant medical achievements of the humankind [1]. It began with the discovery of Salvarsan, one of the first medicines capable of curing an infectious disease—syphilis—without being toxic to the patients. However, it was not until the accidental discovery of penicillin, in 1928, by Alexander Fleming, that research of antibiotics started, reaching its peak between the 1950s and 1960s, a period that became known as the “golden age” [1]. More than 20 new classes of antibiotics were produced between 1930 and 1962, but due to the evolution of new resistant bacteria, discovery of new molecules with antibacterial activity has become even more challenging to the pharmaceutical industry [2,3].

Antibiotic resistance bacteria are one of the main causes of lack of efficacy of antimicrobial agents. Bacterial resistance is caused by modifications in the ability of microorganisms to resist against antibacterial agents either by inactivating them or by causing a decrease in their therapeutic efficacy. Over time, these resistances appear spontaneously in microorganisms due to genetic modifications. The inappropriate use and abuse of antibiotics considerably favors such modifications [3]. This leads to extended infection periods, increased mortality rates, and further economic burden in health systems [4]. Besides the genetic mutations in the microorganism, bacterial resistance can result from the exchange of genetic material among bacteria or phages by: (i) DNA transformation, which is the uptake and incorporation of a DNA fragment; or by (ii) transduction or transfer of bacterial genes through a virus and conjugation, consisting on the transfer of genetic material between a donor and a receptor microorganism [3]. Antibiotic resistance has very diverse mechanisms such a enzymatic mechanisms using by β-lactamases, acetyltransferases or aminoglycoside modifying enzymes [5]. Alteration of membrane permeability preventing the penetration of the antimicrobial agent is also a common resistance mechanism, together with changes in the antimicrobial target (e.g., penicillin-binding proteins or mutations in DNA gyrase and topoisomerase IV) [6].

**Figure 1:**
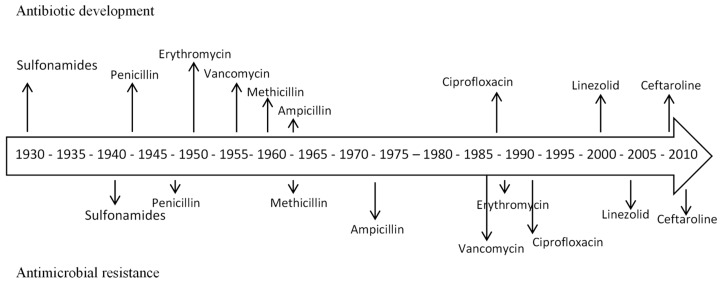
Development of antibiotics and appearance of bacterial resistance over time.

Since the “golden age”, only three new classes of antibiotics active against Gram-positive bacteria, such as methicillin-resistant *Staphylococcus aureus* (MRSA), were discovered and approved: oxazolidinones (linezolid in 2001 and tedizolid in 2014), daptomycin in 2006 (a cyclic lipopeptide) and fidaxomicin in 2011 (a macrocycle drug for *C. difficile*). However, a high number of analogues of existing classes and antibiotic combinations has reached the market [2]. Figure 1 shows the timeline of appearance of antibiotic resistance versus antibiotic development, highlighting the rapid resistance development by bacteria.

Despite the trend for the increasing need of new antibiotics, global disincentives to the use of antibiotics significantly reduced the sales volume when compared to other drugs (e.g., those used in chronic diseases) [7]. In fact, over the recent years, the number of companies involved in the research of new antibiotics has decreased from 25 in 1980 to less than a half remaining Glaxo Smith Kline, Johnson & Johnson, Merck & Co., and Pfizer, while other large pharmaceutical companies have redirected their resources to the development of drugs for chronic diseases and other areas, such as cancer, where the market share is higher and drugs can be marketed at higher prices [8].

In an attempt to reverse this scenario, international authorities have sought to address multi-resistant infection management measures and to promote research and development (R&D) of new therapeutic entities. In February 2017, the World Health Organization published a Global Priority Pathogens List (PPL). Among others, bacterial infections were considered as the greatest concern to public health. For this reason, it is remarkably clear that there is an urgent need for new substances with antibacterial properties. The main objective of this guideline has been to drive researchers to prioritize the R&D of new antibiotics [9]. This report established Gram-negative bacteria as the most critical pathogens for antibiotic R&D, since some strains that cannot be treated with none of the antibiotics currently on the market [1]. In March 2018, DRIVE-AB was created. DRIVE-AB project consists in 15 public partners and 7 private partners responsible for setting guidelines for the rational use of antibiotics, funded by the Innovative Medicines Initiative (IMI). The consortium published a report setting out four incentives considered highly effective in stimulating the pipeline of antibiotics. Grants (for R&D in academic institutions, companies and others), and the pipeline coordinators (governmental or non-profit organizations that track the antibiotic pipeline, identify gaps and actively support R&D projects) are intended to stimulate the early stages of developing and support research groups during the early stages of development. Once the development phase is completed, market entry rewards aim to make the antibiotic market more appealing for investment by giving the companies a reward of, e.g., $1 billion per new antibiotic. These incentives have also been set to fill the low sales volume of new antibiotics. DRIVE-AB has proposed a long-term supply continuity model designed to ensure the continuous supply of essential antibiotics through a series of annual fixed payments to the supplier [10].

These new incentives stimulated the interest of pharmaceutical companies involved in the development of non-traditional drugs, especially nanotechnology industries, which have invested in the development of new nanomaterials identified as promising agents against bacteria resistant to traditional antibiotics.

## 2. Metal Nanoparticles: Overview

Metal-based nanoparticles are the most popular inorganic nanoparticles and represent a promising solution against the resistance to traditional antibiotics. Not only do they use mechanisms of action that are completely different from those described for traditional antibiotics, exhibiting activity against bacteria that have already developed resistance, but they also target multiple biomolecules compromising the development of resistant strains [11].

Metal-based nanoparticles may be characterized by numerous techniques. These methods provide valuable information about their morphology, physicochemical, and electric properties which are crucial for their in vivo activity. The most relevant properties of nanoparticles include aspects as their size, shape, roughness, and surface energy [12].

### 2.1. Metal-Based Nanoparticle General Mechanisms

Bacteria have specific characteristics that explain their behaviour in contact with metal nanoparticles. Since the main toxicological effect induced by antimicrobial compounds in bacteria occurs by direct contact with the cell surface, it is important to understand the differences between the cell wall of Gram-positive and Gram-negative bacteria [12].

Both Gram-positive and Gram-negative bacteria have a negatively charged surface [11]. Gram-positive bacteria have a thick layer of peptidoglycan formed by linear chains alternating residues of N-acetylglucosamine (NAG) and N-acetylmuramic acid (NAM) linked together by a sequence of 3 to 5 amino acids that cross-link each other, forming a cohesive mesh. Additionally, negatively charged teichoic acids (with high levels of phosphate groups) extend from the cell wall to the surface of most Gram-positive bacteria. Gram-negative bacteria, on the other hand, have a slightly more complex structure. In addition to the thin layer of peptidoglycan, Gram-negative bacteria have a phospholipid outer membrane with partially phosphorylated lipopolysaccharides (LPS) that contribute to increase the negative surface charge of their cell envelope [13].

Negatively charged bacterial cell walls attract positively charged nanoparticles to their surface due to electrostatic interactions. On the other hand, positively charged metal-based nanoparticles establish a strong bond with membranes, resulting in disruption of cell walls and, consequently, increase their permeability. In addition, nanoparticles can also release metal ions from the extracellular space, capable of entering the cell and disrupt biological processes [14]. Inside the cell, either metal ions or nanoparticles can induce production of reactive oxygen species (ROS). The oxidative stress generated leads to oxidation of glutathione, thus suppressing the antioxidant defence mechanism of bacteria against ROS. The metal ions are then free to interact with cellular structures (e.g., proteins, membranes, DNA), disrupting cell functions [14]. Metal ions can form strong coordination bonds with N, O, or S atoms which are abundant in organic compounds and biomolecules. Since the bond between metal ions and biomolecules is generally non-specific, metal-based nanoparticles generally exhibit a broad spectrum activity [15].

### 2.2. Synthesis of Metal and Metal Oxide Nanoparticles

Metal-based nanoparticles are not a recent technology. The natural production of metal-based nanoparticles by some microorganisms as a mechanism of heavy metals detoxification has been described. However, the versatility of this technology has only been described over the last decades, with metal-based nanoparticles being widely used in the production of cosmetics and textiles ever since [16]. Their versatility has arisen the interest of the scientific community, which began an endless search for new compositions, applications, and methods of synthesis. Although research has been expanded over the recent years to other less-common metals, the most widely used materials in metal-based nanoparticles include silver, gold, copper, iron, and zinc [17,18,19]. Transition metals are expected to be the best candidates for the synthesis of metal-based nanoparticles since these have partially filled d-orbitals which make them more redox-active (easier to reduce to zerovalent atoms), a feature that facilitates their nanoparticle aggregation [20]. The various synthesis methods developed can be classified as physical methods, chemical methods, and more recently developed biological methods [18].

**Figure 2:**
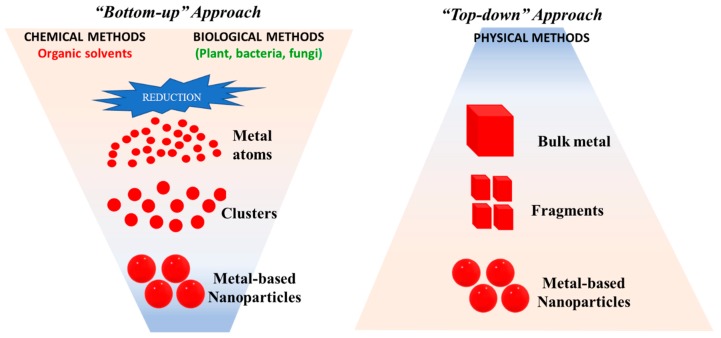
Different methods used for the synthesis of metal-based nanoparticles.

Physical methods use a top-down approach (Figure 2), starting from bulk metal that undergoes fractionation into smaller pieces by mechanical action into successively smaller fragments. Although very simplistic, this technique creates nanoparticles with a fairly dispersed size distribution and is therefore not the most appropriate in the synthesis of metal-based nanoparticles, in which the size is the determining factor for their activity [19]. On the other hand, bottom-up approaches are used in chemical methods involving organic solvents and also in biological methods, which are focussed on green-synthesis processes using different types of microorganisms.

#### 2.2.1. Thermolysis Methods

Generally, this technique relies upon the dissociation of organometallic precursors in organic solvents at temperatures generally higher than 100 °C under inert atmosphere to avoid surface oxidation of the nanoparticles [20]. As a disadvantage of this method, reactions are difficult to apply to large-scale synthesis, due to their highly diluted and exothermic conditions. Otherwise, there are other methods for the synthesis of nanoparticles, such as controlled thermolysis of silver alkyl carboxylates, in order to produce silver nanoparticles (AgNPs) without using organic solvents. As an advantage of this method, the controlled thermolysis can be applied to industrial large-scale synthesis with very low cost [21].

#### 2.2.2. Chemical Reduction Methods

In these methods, a metal precursor dissolved in a solvent is mixed with both a suitable reducing agent and a surfactant in a constantly stirring batch reactor under inert atmosphere. When two or more metal cationic species are present in the solvent, a nanosized phase of variable composition is formed. This constitutes a promising method to obtain metastable metal nanoparticles. The choice of the reducing agent is very wide, but it could be based on the specific redox thermodynamics. Moreover, in the majority of the cases, the activity of reducing agents is strongly dependent on the pH of the solution [22]. For example, for the preparation of copper nanoparticles (CuNPs), the precursor copper acetate is dissolved in stirring deionized water. Hydrazine, the reducing agent, is added to the solution and the nanoparticles are formed afterwards [23].

#### 2.2.3. Biochemical Methods

For these methods, plants, algae, yeasts, fungi, bacteria, and even viruses have been recently used along with chemical reagents [24]. The growth process is undertaken in intracellular or extracellular environment and it relies upon enzymatic or nonenzymatic reduction processes. Gold and silver nanoparticles could be synthesized by bacteria or fungi with a multiplicity of shapes (cubes, triangles, spheres, plates, or wires) according to the specific host cells and method parameters. Despite several patents reporting the use of these methods, biosynthesis process optimization still remains an unsolved problem [25]. 

#### 2.2.4. Electrochemical Methods

Electrochemical methods have demonstrated some additional advantages over chemical methods in the synthesis of size-selective or shape-controlled highly pure metal nanomaterials. A metal sheet is anodically dissolved and the intermediate metal salt formed is reduced at the cathode, giving rise to metallic particles stabilized by ammonium salts [26]. Some authors reported the synthesis of bimetallic Cd-Ag nanoalloys by sequential electrodeposition of two different cations on a carbon electrode [27]. Similarly, palladium metallic nanostructures were obtained via templated-assisted electrodeposition from electrolytes containing salts of the relevant cation precursor [28]. Other authors reported the synthesis of AuNPs via direct electroreduction of gold ions bulk by utilizing polyvinylpyrrolidone (PVP) in enhancing the gold nanoparticle formation and inhibiting the metal deposition on the cathode [26].

#### 2.2.5. Wave-Assisted Chemical Methods

Sonochemical methods rely upon the use of a source of ultrasounds inducing cavitation in a solution containing a metal precursor mixed with a reducing agent and a surfactant as stabilizer. The formation and further implosion of microcavities in the liquid phase produce local spots with extremely high temperatures (theoretically higher than 3000 °C) that may trigger chemical reactions otherwise unfeasible with traditional techniques [29]. In radiolytic processes, a metal precursor mixed with a suitable reducing agent is subject to an electromagnetic or particle irradiation, such as an accelerated electron beam [30], gamma-rays [31], X-rays [32], and ultraviolet rays [33]. AgNPs can be prepared by ultrasonic wave assisted synthesis, by reducing AgNO_3_ with strong reducing agent as sodium borohydride in the presence of ultrasonic waves. A greyish precipitate is formed, which is irradiated ultrasonically and then centrifuged, obtaining the AgNPs [34].

Over the last years, micro-wave assisted synthesis has been considered as an eco-friendly and fast method. This fact is because the stabilizer and complexing agent can be replaced by less polluting materials, such as chitosan and polymers. Moreover, this method is able to carry out chemical transformations in minutes. For example, AgNPs can be also prepared by complexing PVP and reducing Ag^+^ ion with *N*,*N*-dimethylformamide [35].

#### 2.2.6. Cementation Methods

When a strongly electropositive metal A (sacrificial element) is left in contact with a solution containing ions of a less electropositive metal B, the following spontaneous reaction is thermodynamically allowed, and metal B separates in elemental form

A + n/mBm+ = An+ + n/mB(1)

This reaction, which is commonly used in industry to purify solutions in hydrometallurgy, can be used to reduce cations obtaining metal nanoparticles or aggregates with a relatively simple and cheap process [36]. The two main disadvantages of this method are the poor control of nanoparticle agglomeration owing to a sticking of the cemented metal phase B on the surface element A. However, if A contains impurities, they can contaminate B as a consequence of a multicluster surface etching of A. These problems can be avoided by damped mechanically with a tailored hydrodynamic control [37]. For example, CuNPs can be synthetized with a reduction of copper from a copper nitrate salt in the presence of iron, and to prevent the formation of larger sized CuNPs, the sample was continuously ultra-sonicated. The obtained nanoparticles sizes were recorded between 90 and 150 nm [38].

#### 2.2.7. Biological Methods

Biological methods arose from the need to develop new more environmentally friendly techniques that exclude the use of organic solvents and toxic chemicals (Table 1). They also proved to be safe and economically sustainable alternatives. Critical aspects of the synthesis of metal-based nanoparticles, such as size distribution and crystallinity, can be overcome for example by selecting the strain, incubation temperature and time, concentration of metal precursor, and optimal pH conditions [39].

Biological methods take advantage of the defence mechanisms present in specific organisms (against high concentrations of metal ions) to produce metal-based nanoparticles. These methods include intracellular (e.g., bioaccumulation) or extracellular mechanisms (e.g., bioabsorption, biomineralization, complexation or precipitation) [55].

The use of fungi in the production of metal-based nanoparticles offers advantages for industrial scale production when compared to bacteria since these organisms have a higher resistance against the flow pressure and agitation of the bioreactors [56]. However, in recent years, most of the studies report the use of plant extracts because, in addition to the advantages mentioned above, its use facilitates the treatment of samples, the scale-up production, and the collection of the product of interest.

## 3. Silver Nanoparticles (AgNPs)

For a long time, silver has been used as an antimicrobial agent for wound healing, both in its solid state and with salt solutions to clean wounds. Nowadays, dressings impregnated with AgNO_3_ can be found [57]. Silver exhibits very interesting properties due to its chemical stability, good conductivity, catalytic, and antibacterial activity. Moreover, nanoparticles made of silver (silver nanoparticles, AgNPs) are one of the most widely studied nowadays [58]. AgNPs have been applied in different fields such as textile, cosmetics, food industry, and biomedicine. In the biomedical field, they are gaining strength especially due to their applications as antimicrobial agents, as coating for medical devices, and as carrier for chemotherapeutic drugs [59]. Despite being widely studied, continuous research towards the development of more bio-sustainable synthesis methodologies is still needed, together with the disclosure of mechanisms involved in the toxicological effects AgNPs.

### 3.1. Synthesis

#### 3.1.1. Conventional Chemistry

Compared to other methods, chemical synthesis of AgNPs is relatively cheap and easy to implement at a large scale while maintaining a monodispersed size distribution.

**Figure 3:**
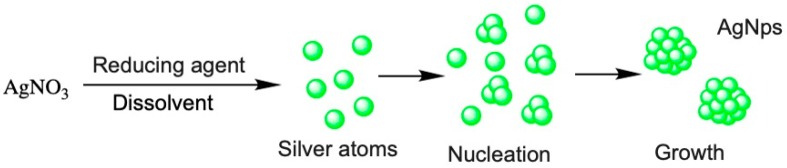
Process for the synthesis of AgNPs.

Among the variety of chemical methods available for the production, chemical reduction is the most widely used for this type of nanosystems. This process employs the use of three main components, (i) a metal precursor, (ii) reducing agents, and (iii) stabilizing agents [60]. Basically, two stages of nucleation and growth are involved (Figure 3). In this synthesis, the stabilizing agent can have a dual function, i.e., also acting as a reducing agent in the same reaction [61].

Appropriate average size, polydispersity, and shape of AgNPs can be achieved by controlling the nucleation stage, i.e., by monitoring the experimental parameters, such as the precursor used in the reaction, reducing agents, reagent concentration, pH, and temperature [62,63,64,65].

A critical step in the synthesis of AgNPs is their stabilization, especially in order to prevent agglomeration and oxidation processes. Therefore, one of the most common strategies is the use of stabilizing agents that are capable of protecting AgNPs. For the stabilization, chitosan, amine derivatives, thiols, or gluconic acid can be used. In addition, it has also been proven that the use of polymeric compounds, such as polyvinylpyrrolidone (PVP), polyacrylates, polyvinyl alcohol (PVA), polyacrylonitrile, polyacrylamide, or polyethylene glycol (PEG), is also useful. Finally, stabilization can be achieved by electrostatic repulsion by incorporating a negative charge on the surface of these NPs mainly through citrate groups [62].

Among all the chemical methods to obtain AgNPs, the Creighton method is the most widely used because it allows to produce monodispersed and small size (around 10 nm) nanoparticles [63]. In this method, the precursor agent is AgNO_3_ and the reducing agent is NaBH_4_. The reaction that is carried out is as follows:

2 AgNO_3_ + 2 NaBH_4_ → 2 Ag_(s)_ + B_2_H_6_ + H_2_ + 2 NaNO_3_ (2)

#### 3.1.2. Green Chemistry

**Figure 4:**
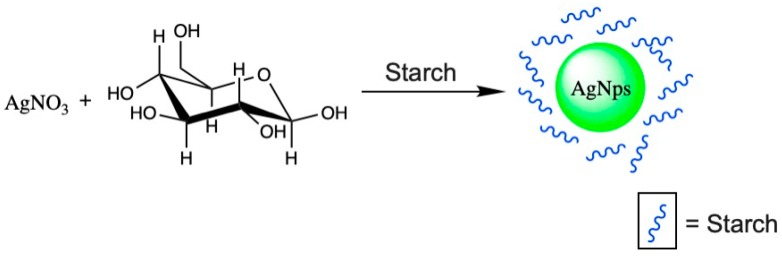
Chemical reduction of AgNO3 salt from β-D-glucose.

Although chemical synthesis has the advantages of being of low cost and of high performance, the use of reducing agents is harmful. Therefore, methods that use environmentally friendly reagents have been developed. Special interest is the method using β-D-glucose as a reducing agent to cause the chemical reduction of AgNO_3_ salt. This method employs starch as a stabilizing agent (Figure 4**).** These green synthesis is able to obtain AgNPs under 10 nm of mean size [64].

Ho et al. described a synthesis method using ascorbic acid as a reducing agent for the reduction of AgNO_3_. They were able to obtain hybrid AgNPs inside a polylysine shell modified by different fatty acids. This green synthesis method was able to develop AgNPs of mean size between 2 and 5 nm [65].

Nowadays eco-friendly methods of biosynthesis are being gradually replacing the traditional chemical synthesis with increasing publications about this topic in the last years. The biosynthesis of AgNPs uses the bases of chemical synthesis, but instead of a chemical entity, this takes advantage of the reductive properties of biological entities.

The biosynthesis of AgNPs by bacteria able to produce reductase enzymes results from an intra- or extracellular process [66]. The intracellular biosynthesis uses facilitated transport of Ag^+^ ions into the bacterial cell. Bacteria can also produce AgNPs by transforming ionic Ag^+^ to neutral Ag^0^. In this case, additional step for recovery of AgNPs (as cell lysis) is required. Extracellular biosynthesis occurs outside bacterial cell either using bacterial biomass, the supernatant of bacterial cultures, or cell free extracts. An organic base is used in the supernatant to ensure the proper recovery of AgNPs by centrifugation and further resuspension [67]. Extracellular biosynthesis is preferred over the intracellular process as it does not require downstream processing one of the first bacteria used for the synthesis of AgNPs by culturing in high concentrations of AgNO_3_ was *Pseudomonas stutzeri* AG29, a silver-resistant bacterium isolated from a silver mine which reduces Ag^+^ to Ag^0^ with accumulation inside the cell [68]. Usually, AgNO_3_ is added to the organic base, and the mixture is incubated at optimized conditions [69]. The ionization of AgNO_3_ is described in the equation
AgNO_3_(aq) 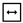 Ag^+^(aq) + NO_3_^−^(aq)(3)

Nitrate reductase is an enzyme produced by some bacteria activated at alkaline pH in the presence of a substrate (NO_3_^−^). This enzyme catalyses the reduction of nitrate to nitrite, illustrated in the equation [67]

Ag^+^(aq) + NO_3_^−^(aq) + NADH + H^+^ + e^−^ → Ag^0^(s) + NO_2_^−^(aq) + NAD^+^ + H_2_O(l) (4)

Jang et al. described that alkaline pH improves the yield of the reaction, also producing smaller sized particles in the pH range of 8 to 10 [70]. On the other hand, at acidic pH, Ag^+^ ions precipitate and AgNPs synthesis is hardly observed [70].

Fungi produce extracellular enzymes which are secreted outside the cell and are responsible for extracellular digestion of macromolecules followed by absorption of nutrients. This unique characteristic gives them great relevance in extracellular synthesis of AgNPs. After incubation and growth of colonies, fungi are usually separated from the aqueous medium containing extracellular enzymes. The first ones are discarded and AgNO_3_ is added to the medium. This mixture is then incubated usually at temperatures close to room temperature. Production of AgNPs follows the equations described above. The reaction of synthesis can be confirmed by change in the color of the medium [71]. Several aspects as medium properties, incubation time and temperature, AgNO_3_ and biomass concentrations and activity, are of crucial relevance. Zhao et al. evaluated the optimization of extracellular biosynthesis of AgNPs by fungi, being, pH 7, 25 °C, 1 mM AgNO_3_, and 15–20 g of wet cell filtrate the optimal conditions. The obtained nanoparticles were of spherical shape with a mean size recorded between 25 and 30 nm [72].

Synthesis of AgNPs using plant extracts is based on their relatively high levels of steroids, sapogenins, carbohydrates, and flavonoids, that act as reducing agents, as well as bio-capping compounds reducing agglomeration of nanoparticles and allowing a better size control [73]. In general, the obtention of AgNPs from plant extracts is a simple process. The freshly collected plant parts are cleaned with sterile water, dried in the shade, and powdered. For the preparation of the plant extract, the dry powder is boiled in deionized water. The resulted infusion is filtered until no insoluble material is present. A certain amount of plant extract is then added to the solution containing 1 mM AgNO_3_. AgNPs synthesis reaction may again be checked by a color change of the medium (usually to dark brown) and confirmed with the ultraviolet-visible (UV–Vis) spectra. AgNPs formed may be easily collected by repeated centrifugation processes at 12,000 rpm for 15 min [73].

#### 3.1.3. Physical Methods

There is a wide variety of physical methods for the synthesis of AgNPs, but evaporation/condensation is one of the simplest and best controlled. Moreover, laser ablation is also a method that allows to obtain a large number of nanoparticles in a short time. They are also the most widely used physical techniques. However, these methods are of high cost among other disadvantages, as the need to use a tubular furnace (which takes up a lot of space and consumes a large amount of energy) and the increasing temperature during the process requiring a long time to achieve thermal stability [60].

### 3.2. Characterization of AgNPs

Numerous methods have been used to control and characterize AgNPs. The most often reported are UV–Vis spectrophotometry, X-ray diffractometry (XRD), transmission electron microscopy (TEM), and infrared spectroscopy (IR). 

UV–Vis spectrophotometry is usually performed at various time intervals during the reaction and provides valuable information about the success of AgNPs synthesis [74]. AgNPs have an extraordinary efficiency to absorb and disperse the light. This interaction with the light is produced because the electrons of the metal surface, when excited, experience a collective oscillation by characteristic wavelengths. These oscillations are known as the surface plasmon resonance. In the case of AgNPs, this appears around the wavelength of 400 nm; its exact position depends on the diameter, shape, and distribution of the nanoparticles [75].

XRD evaluates the crystalline nature of AgNPs, recorded in the 2θ range of 30–80° and provides confirmation about the morphology. XRD pattern of AgNPs usually reveals the formation of face-centred cubic (FCC) structures of metallic silver. The prominent peaks are observed at 2θ = 38.19°, 44.46°, 64.63°, and 77.34°, and correspond to the (111), (200), (220), and (311) planes, respectively [76].

Alternatively, the Debye–Scherrer equation allows for an approximate calculation of the average size as

(5) $$D=\frac{K\lambda }{\beta \cos\theta }$$

where *D* is the size (nm), *λ* is the wavelength of radiation (nm), *β* is the full width at half maximum (radians), and *θ* is the half of the Bragg angle (radians) [77].

TEM image allows the visualization of biosynthesized AgNPs morphology. Usually, AgNPs are spherical with a quite dispersed average size [76].

IR is usually carried out to identify key functional groups and to characterize biomolecules bound specifically on the synthesized AgNPs either by free amine groups, cysteine residues, or electrostatic attraction of carboxyl groups [78]. The biologically synthesized AgNPs are mixed with potassium bromide to make a pellet that is placed into the sample holder. Based on analysis, its assumed that these biomolecules and proteins may be involved in the capping stabilization. IR spectra vary with the characteristics of the organism/specie used in the AgNPs synthesis [71]. 

Other procedures include scanning electron microscopy (SEM) and atomic force microscopy (AFM) also provide information on the size and size distribution. In addition, dynamic light scattering (DLS) determines size and polydispersity index and electrophoresis laser doppler is used to measure zeta potential (as an indirect measure of the surface electrical charge) of the nanoparticles. Moreover, field emission scanning electron microscopy (FESEM) provides information on the surface morphology, while energy-dispersive X-ray (EDX) is used for elemental analysis or chemical characterization.

Zeta potential measurement is a valuable tool for determination of AgNPs stability and surface electrical charge of the aqueous colloidal suspensions. Balakrishnan et al. included zeta potential determination in their extensive AgNPs characterization which was determined to be about −9.56 mV, indicating a slight repulsion of AgNPs [79]. Farhadi et al., on the other hand, recorded a value of about −35 mV. Since this latter value is higher than |30| mV stability of the colloidal AgNPs suspension is ensured, illustrating the repulsion between synthesized nanoparticles which prevents agglomeration phenomena [80]. Zeta potential values can be either positive or negative, however, its value is frequently negative probably due to the possible capping of bio-organic components present in the extract. AgNPs of negative zeta potential are usually associated with less antimicrobial activity [81].

### 3.3. Pharmacokinetics

In general, biological membranes possess selective permeability allowing the entry of some biomolecules, mostly small and lipophilic molecules and metal ions. However, AgNPs pharmacokinetics has been shown to be dependent on various factors such as dose, exposure route, species, and gender of the tested organisms [82]. In order to determine these parameters, Bachler et al. developed a physiologically based pharmacokinetic model (PBPK) for humans to provide an evaluation about the exposure and risk assessment of AgNPs. However, to keep this PBPK models as simple as possible, they considered ADME (absorption, distribution, metabolism, and excretion) of AgNPs followed a first-order kinetic [83].

### 3.4. Absorption

There are four potential administration routes for AgNPs in mammals. These include oral, dermal, pulmonary, or intravenous. As new promising agents against bacterial infections, AgNPs are being developed not only for topical, but also for systemic infections. For this reason, it is important to determine their pharmacokinetic parameters. Absorption of AgNPs through biological membranes depends on their physicochemical characteristics, as size and shape.

#### 3.4.1. Gastrointestinal Absorption

In case of oral administration and during gastrointestinal absorption, AgNPs undergo a series of changes triggered by high temperatures, variable pH, changes of saline balance and enzymes in the gastrointestinal tract. After gastric digestion, the number of nanoparticles drop significantly, rising back to original values after intestinal digestion. Reduction in number of particles is caused by their clustering promoted by the chloride present in the stomach. During intestinal digestion, these clusters are disintegrated back into single AgNPs. This phenomenon is believed to be caused by the increase in pH from gastric to intestinal medium. Results like these indicate that, under physiological conditions, AgNPs reach the intestine mostly in their original form [84].

Although most of AgNPs reach the intestine, its gastrointestinal absorption rates are extremely low, as proved by Bachler and colleagues, who concluded that after oral administration of AgNPs, the intestinal absorption fraction was about 0.12% to 0.88% in humans and ionized silver (Ag^+^) presented a fraction of approximately 5% in rats [83]. The low absorption of AgNPs after an oral dose may be associated with the binding of nanoparticles to non-digestible food components resulting in a higher fecal excretion of silver [85].

#### 3.4.2. Pulmonary Absorption

Absorption from the alveoli to the bloodstream is mostly dependent on size of the nanoparticles. While ultrafine AgNPs dissociates rapidly and silver spreads to the blood capillaries, larger or agglomerated AgNPs are retained in the lungs. The remaining AgNPs in the alveoli are rapidly eliminated through local macrophages phagocytosis [86].

In order to determine absorption fractions of AgNPs, Bachler et al. used as reference the concentrations needed to cause argyria (a condition that occurs after prolonged exposure to silver and is characterized by irreversible purple to grey coloration of skin/eyes) after pulmonary or intestinal uptake. According to these authors, this value can be up to 3.75 times higher to an oral dose when compared to an inhaled dose, which suggests a more extensive lung absorption. During their experiments, Bahler et al. obtained absorption fractions of 20.1% for inhalation and 3.25% for oral absorption, which are consistent with the values referred above [86].

#### 3.4.3. Cutaneous Absorption

The first route for dermal penetration involves transport through the stratum corneum, either by transcellular (diffusion through the cells) or intercellular pathway (diffusion through the gaps between corneocytes). The second route for epidermal penetration includes the entry via skin appendixes as hair follicles and sweat glands [87].

AgNPs, as other metallic nanoparticles, accumulate in the follicle where they form a deposit which cannot be removed by natural desquamation or body washing. These deposits allow a gradual absorption of nanoparticles from the follicles to the blood capillaries [88]. According to Larese et al., AgNPs can penetrate intact human skin. However, the amount capable to penetrate in compromised skin has been reported to be 5 times greater than in the intact skin [89].

### 3.5. Distribution

Accumulation of AgNPs in the tissues is dependent on the administration route, e.g., high levels in skin after dermal administration or in the lungs during pulmonary administration. After oral administration, AgNPs reach high concentrations in stomach and in small intestine. After oral administration and absorption, silver undergoes the first-pass metabolism with further excretion in the bile, reducing systemic distribution in the body tissues [90].

After intravenous injection, concentration-time curves revealed a rapid decline in silver concentration during the first 10 min which indicates a fast distribution of AgNPs to tissues followed by stabilization. Although silver distributes to all organs, it achieves higher accumulation in the spleen and liver after a single dose injection. Prolonged treatments result in a slight decrease in liver accumulation with AgNPs depuration and excretion through the bile and a redistribution of silver to other organs as kidney, heart, lungs, testes, and brain [91].

### 3.6. Metabolism and Excretion

Stabilization of AgNPs by proteins adsorbed onto their surface blocks dissolution of silver making impossible the formation of new soluble silver species. Instead, stable AgNPs undergo direct complexation into silver sulphide particles which accumulate in the tissues [83]. Activation of metallothioneins in the liver may also be pointed out as a reason for AgNPs accumulation in this organ. These small proteins that bind in a variable size of 7 KDa present a high number of thiol groups which are involved in detoxication of heavy metals in liver, but also in other organs as kidneys, intestine, and brain [91].

The elimination of AgNPs is slow. The fraction of AgNPs excreted by the kidneys can be considered negligible, being less than 0.01%, while biliary excretion is the main route of silver clearance, being responsible for more than 50% [92]. This route includes the complexation of silver with glutathione (GSH) generating silver-GSH complexes [83]. However, elimination will be governed by the tissue where the AgNPs are found, and also by the dose and the particle size. As such, AgNPs have shown an elimination half-life that will vary from 29 days to more than 260 days after oral administration [93].

### 3.7. Antimicrobial

Despite several approaches that have been made over the years, the precise mechanism of action of AgNPs is still not fully understood. The antimicrobial action of AgNPs is linked to four main mechanisms: (i) attraction to bacterial surface, (ii) destabilization of bacterial cell wall and membrane with change in its permeability, (iii) induction of toxicity and oxidative stress by generation of ROS and free radicals, and (iv) modulation of signal transduction pathways [62].

Adhesion of AgNPs onto the surface of bacteria is described by many authors as the first step of a complex mechanism of bacterial inhibition. AgNPs adhesion is highly influenced by their size, but also by their zeta potential. Depending on the method for their synthesis, AgNPs may have a positive, neutral, or negative surface charge. Abbaszadegan et al. demonstrated that by varying the surface charge of nanoparticles, a marked fluctuation of the antibacterial activity occurs. Since bacterial surface shows a slightly negative charge, positively charged AgNPs are strongly attracted to the surface of the bacteria, resulting in increased antibacterial activity (Table 2). On the other hand, neutral or negatively charged nanoparticles have a significantly decreased antibacterial effect. However, an increase in the concentration of AgNPs allows the attenuation of electrostatic repulsion through a bacterial surface saturation method [94].

**Figure 5:**
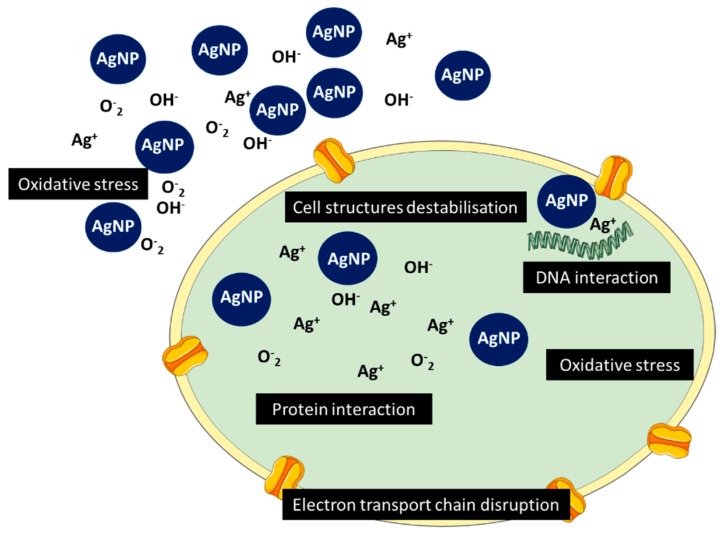
Schematic representation of AgNPs mechanism of antimicrobial activity.

After adhesion onto the bacterial surface, AgNPs can interact with the cells via two different mechanisms. Smaller AgNPs penetrate directly into the cell, while larger nanoparticles are retained outside the bacteria. In both cases, AgNPs continuously release Ag^+^ ions. These ions bind to cell membrane structures destabilizing the membrane potential and causing proton leakage. Cell wall destabilization highly increases bacterial permeability, allowing larger AgNPs to enter the cell [95]. Once inside the cell, AgNPs and Ag^+^ ions interact with numerous structures and biomolecules as proteins, lipids, and DNA, resulting in cell dysfunction. AgNPs are well known by their high capacity to produce reactive oxygen species (ROS) and free radicals as hydrogen peroxide (H_2_O_2_), superoxide anion (O_2_^−^), and hydroxyl radical (OH^•^). Although ROS occur naturally in bacteria as a result of cellular respiration, under normal circumstances bacteria have defense mechanisms—such as glutathione (GSH), superoxide dismutase, and catalase—that act as antioxidant enzymes and eliminate these toxic species. High concentrations of Ag^+^ released by AgNPs produce extreme levels of oxidative stress (Figure 5). Even though antioxidant enzymes remove some of the released ions, these are not enough to neutralize the AgNPs amount [96]. These species interact with respiratory chain proteins on the membrane and inactivate enzymes due to their high affinity to phosphates, thiol, and carboxyl groups [97]. Their link to phosphate groups inhibits phosphorylation of proteins which is frequently involved in enzymatic activation, ultimately resulting in inhibition of bacterial growth. Dephosphorylation of tyrosine residues of protein was also been implicated in disruption of biosynthesis and transport of exopolysaccharide and capsular polysaccharide to the membrane, thereby disruption of cell cycle [62]. Additionally, Ag^+^ can intercalate DNA strands forming complexes with nucleic acids between the purine and pyrimidine base pairs, disrupting H-bonds between them [96].

### 3.8. Other Pharmaceutical Properties

Bactericidal properties of AgNPs are the most widely studied, but a wide variety of other biomedical properties—such as antifungal, antiviral, antiamebial, anti-cancer, anti-angiogenic, and anti-inflammatory activity—are also being exploited [59]. New antifungal agents are also a demand, in particular, for immunosuppressed patients. AgNPs have a high antifungal potential, for example in *Candida albicans* infections, AgNPs stabilized with dodecyl sulphate show a better activity than conventional treatment [98]. AgNPs also show antiviral properties. In the case of activity against HIV-1 AgNPs have demonstrated anti-retroviral ability in addition to a potent virus inhibition effect. They have also proven to be efficient inhibitors against hepatitis B virus (HBV) [99]. When associated with drugs capable of crossing the blood–brain barrier such as diazepam, AgNPs also showed antiamebial activity [100].

Also, due to the cytotoxic effects of AgNPs, they are seen as a promising alternative in cancer therapy. AgNPs have been used against breast, hepatocellular, or lung tumors, but also as carriers of anticancer drugs in chemotherapy. Faedmaleki et al. showed that AgNPs are able to exhibit a 44-fold inhibitory effect on a HepG2 liver cancer cell line with an IC_50_ of 2.8 ppm (µg/mL) compared to a non-carcinogenic cell line with an IC_50_ of 121.7 ppm [101]. AgNPs demonstrated to be antiangiogenic and antiproliferative [59]. AgNPs also showed anti-inflammatory properties by inhibiting pro-inflammatory cytokines [102].

### 3.9. Toxicity Assessment

The toxicity of AgNPs is directly related to their physicochemical properties such as surface charge, solubility, shape, size, specific surface area, and agglomeration status. As AgNPs have high surface area when compared to bulk counterparts, their reactivity in biological media is also high, with higher risk of toxicological events [103]. AgNPs with a negative surface charge have shown lower toxicity than positively charged nanoparticles [104]. Similarly, when AgNPs dissolve and lose their spherical structure, their toxicity increases. However, further studies are required to assess whether the toxicity of AgNPs is produced by the nanoparticles themselves or by the silver ions that are released. The shape is also a relevant element to assess the toxicity as there are more toxic forms of AgNPs than other types of nanoparticles, being nanospheres with less toxicity.

It has been firstly assumed that AgNPs would be less toxic to mammalian cells than to bacterial cells [121]. It has been however demonstrated that AgNPs have no such selectivity. AgNPs also induce toxicity to mammalian cells as demonstrated in different studies showing toxicity in hepatocytes and neuronal cells [122]. Toxicity of AgNPs to humans is caused by different mechanisms. On one hand, it seems to be associated with the oxidative nature. Due to their interaction with proteins and enzymes with thiol groups (in superoxide dismutase) which are key in the antioxidant defence mechanisms of cells. On the other hand, AgNPs are able to stimulate DNA damage, due to their genotoxic potential, breaking DNA chains and causing chromosomal aberrations [123]. Regarding aquatic media, some studies have been reported. Harmon et al. reported the kinetics of AgNPs of different sizes. They have shown that the aqueous media with high conductivity increase the risk of AgNPs aggregation and decrease toxicity. Toxicity values were greater for 20 nm rather than nanoparticles between 50 and 80 nm [124]. Moreover, using a green alga model (*Chlamydomonas acidophila*) it has been reported that after 24 h of exposure, the chlorophyll content and cellular viability of the alga decreased significantly and their ROS production increased proportionally to the concentration of AgNPs [125]. Concentration-dependent toxicity has also been observed in zebra fish, causing an increase in mortality rates [126]. In this organism, AgNPs were able to induce cardiac and morphological abnormalities leading to death in exposure to high concentrations [126].

The bioavailability of AgNPs depends on the soil [127]. AgNPs are less retained in glass beads, quartz or artificial soils while they are easily retained in natural soils [128].

## 4. Copper and Copper Oxide Nanoparticles (CuNPs, Cu_2_ONPs and CuONPs)

Cooper is a semiconductor material considered to be an excellent candidate for the synthesis of metal-based nanoparticles. Besides being highly resistant to heat, it is also robust, stable, cheap and easily synthesized [94,95].

### 4.1. Synthesis

CuNPs and CuONPs can be synthesized by various processes. Among all, biocompatible processes emerged as the most investigated in the past few years. Independently of the selected method, during synthesis, CuSO_4_, CuCl_2_ · 2H_2_O, Cu (NO_3_) _2_ or Cu (CH_3_COO)_2_ are the most frequently used copper precursors. Synthesis of CuONPs is divided in two steps illustrated below [95]:

Cu^2+^ + 2e^−^ ^reduction^ Cu0 + Air (O_2_) ^oxidation^ CuO (6)

The first reaction consists in the reduction of the ionic precursor (Cu^2+^) with formation of Cu^0^ which is highly unstable in the presence of oxygen, and for this reason, it is oxidized to CuO. Occurrence of the reaction may be assessed by observing a color change of the solution: bleaching of the solution with Cu^0^ formation which changes again, usually to a brownish tone after the conversion to CuO [95].

### 4.2. Pharmacokinetics

Since copper is an essential microelement present in all tissues in the human body, its distribution and accumulation are hard to assess [96]. For this reason, to date no work has been published regarding pharmacokinetics of CuONPs. However, a study reported pharmacokinetics of Cu^2+^ and Zn^2+^ after an intravenous dose of a drug containing these elements in rats. The authors determined the baseline copper concentration in blood (1.09 ± 0.04 mg/mL) by subtracting basal values from total metal levels. From the obtained results, copper is distributed to all tissues having affinity to specific transporters that will mediate its entrance in the cells. Similarly, mammalian cells possess Cu-efflux transporters that control the excess of Cu in the intracellular medium, maintaining copper homeostasis [97,98].

### 4.3. Pharmacodynamics

It has been suggested that CuNPs have a greater ability to inhibit bacterial growth because of their direct contact with bacterial cells (attributed to better electron transfer between bacteria and CuNPs). In this case, the slightly negative bacteria and the metallic nanoparticles act as electron acceptors, both contributing to the electron transfer and rupture of the bacterial membrane. Moreover, light irradiation can lead to excited electron-holes pairs in CuO, which show that the bacteria inactivation could also be due to a photocatalytic process. In addition, antibacterial characteristics displayed by copper are a result of cellular damage after contact between released Cu^2+^ ions and bacterial membrane.

However, CuNPs are more instable and have high susceptibility to oxidation. This problem was overcome by the conversion of CuNPs to CuONPs, the latter having greater stability but with slightly less activity [99].

### 4.4. Pharmaceutical Properties 

CuONPs have shown antimicrobial effects (Table 3). The mechanism of antibacterial activity of CuONPs is not well elucidated yet, but it is believed that it involves bacterial cell wall adhesion triggered by electrostatic interactions. Dissociation of Cu^2+^ induces the generation of ROS that contact with cellular membranes. These ions also have the capacity to enter the cell, causing membrane damage which is associated with disruption of cells internal content and bacterial cell leakage [11,99].

Moreover, their antibacterial activity has been studied particularly against microorganisms such as *E. coli*, *V. cholera*, *P. aeruginosa*, *S. typhus*, *S. aureus. E. faecalis*, *B. subtilis* and *S. faecalis* [100,101]. Mirhosseini confirmed in vitro that CuONPs had antibacterial properties, reducing significantly the growth of *S. aureus* and *P. aeruginosa* at the concentration of 500 μg/mL and also showing growth reduction percentages by 24% for *S. aureus* and 7.9% for *P. aeruginosa* [129]. Yoon et al. examined the antimicrobial effect of copper nanoparticles which reduced by 90% *E. coli* and *B. subtilis* at the concentrations of 33.49 μg/mL and 28.20 μg/mL, respectively [130]. Kumar et al. also synthesized colloidal CuONPs from glucose, starch and CuCl_2_ and its antibacterial properties were assessed against *E. coli* (Gram-negative), *S. epidermis* (Gram-positive), a methicillin resistant *S aureus* (superbug MRSA) isolate and the spore-forming *Bacillus megatherium*. Results showed that treatments of CuONPs, with elemental copper concentrations of 0.0113 and 0.00113 113 mol L^−1^ kill all the microorganisms assessed [131].

Moniri et al. synthesized ultra-small CuONPs obtaining a minimum inhibitory concentration (MIC) (derived from both precursors) against *E. coli* and *S. aureus* of 3.75 and 2.50 mg/mL, respectively [132]. Ishaque and Kannabiran biosynthesized CuONPs which showed inhibited the bacterial pathogens B. cereus, *P. mirabilis* and *A. caviae* even at 5 μg/mL concentration [133].

### 4.5. Toxicity Assessment

Few studies have addressed the biosafety effects of CuNPs on the embryogenesis of vertebrates. Zhang et al. observed that CuNPs can develop mental abnormalities in zebrafish embryos (tail and spinal cord flexure and truncation, yolk sac edema and fin abnormality, head and eye hypoplasia, and no swim bladder and reduced digestive gut) treated even with 0.15 mg/L (2.3 μM) concentration. High mortality was observed in embryos treated with 0.5 mg/L (7.8 μM) CuNPs, and the mortality increased in a dose-dependent manner. Also, death was observed in embryos treated with 1 mg/L (15.6 μM) CuNPs even at 24 h post-fertilization [134]. In the same way, Yet et al. investigated the toxic effects of CuNPs on lateral-line hair cells of zebrafish embryos. CuNPs were found to cause toxic effects in a dose- dependent manner. Values of the 96 h 50% lethal concentration (LC50) of CuNPs were 2.61 ppm (41.1 μM). Embryos were unable to survive at ≥5 ppm (78.8 μM) of CuNPs and the number of FM1-43-labelled hair cells and the microstructure of hair bundles was significantly impaired [≥0.01 ppm (0.16 μM)] [135]. On the other hand, in vitro air–liquid interface studies, provide data on nanotoxicity of metal oxides. Therefore, Jing and colleagues evaluated the toxicity of CuONPs in human bronchial epithelial cells (HBEC) and lung adenocarcinoma cells (A549 cells). They found that CuONP exposures significantly reduced cell viability, increased lactate dehydrogenase (LDH) release and elevated levels of reactive oxygen species (ROS) and IL-8 in a dose-dependent manner [136].

## 5. Gold Nanoparticles (AuNPs)

AuNPs are colloidal or clustered particles composed of a gold core, an inert and biocompatible compound [137]. One of the advantages of these particles are their synthetic versatility, which allows the control of their size, shape and surface properties. Furthermore, their coating can be modified to control particle solubility, stability and interaction with the environment. Also, the particle surface can bind thiols and amines, providing functional groups to the AuNPs for labelling, targeting and conjugating pharmacologic molecules [138]. Their unique characteristics make them a material of extreme interest in the medical field due to their optical and electronic properties of Au. Some of the major areas of application of AuNPs include biosensors and bio-imaging, drug delivery systems, and also the treatment of some cancers. Meanwhile, some researchers have been interested in the potential antibacterial activity of AuNPs since this material is less toxic to mammalian cells compared to AgNPs, the most common nanoparticles employed as antimicrobial agents (Table 4) [40].

### 5.1. Synthesis

There are several methods for the synthesis of AuNPs, including chemical, physical and biological pathways, all of them are based on the reduction reaction of chloroauric acid (HAuCl_4_) followed by agglomeration in the presence of a stabilizing agent [139].

AuCl^−^_4_ + 3e^−^ ^reduction^ Au^0^ + 4Cl^−^ (7)

The reaction is easily detected by the color change of the solution from pale yellow to pinkish red color caused by the alteration in the surface plasmon resonance of the newly formed AuNPs. This is due to the fact that at nanosize, the surface electron cloud of gold vibrates, absorbing the electromagnetic radiation of a certain wavelength. In most cases, a peak absorption of AuNPs between 500 and 600 nm (~521 nm) is observed [40].

### 5.2. Pharmacokinetics

AuNPs biodistribution is dependent on many variables, including size and geometry of nanoparticles, surface chemistry and type of stabilizing agent [140].

Although AuNPs distribute through all the organs, they present a size-dependent distribution pattern with smaller nanoparticles showing a more widespread distribution. AuNPs remain in the body for long periods of time, being eliminated very slowly in the faeces and urine. 

After intravenous injection, AuNPs are preferably accumulated in some organs, mostly in the liver, followed by spleen and lungs. Their biodistribution presents a first phase of distribution followed by a second phase of redistribution and elimination. Balasubramanian et al. described a redistribution time of one month after intravenous injection in rats. During this period, they observed an increase in the levels of gold in the kidneys, testis and blood along with persistently high levels of gold in the liver, spleen and adrenal glands [141].

### 5.3. Pharmacodynamics

AuNPs are active against Gram-negative and Gram-positive bacteria, namely *E. coli*, *P. aeruginosa*, *S. typhi*, *Serratia sp*, *K. pneumoniae*, *S. aureus*, *B. subtilis* and *E. faecalis*, among others (Table 3). The fact that AuNPs are relatively inert implies that they exhibit no apparent intrinsic antibacterial activity. Thus, it is understood that their main mechanism of bacterial toxicity is based on direct adherence of AuNPs onto the bacterial surface driven by electrostatic forces. This mechanism is highly dependent on nanoparticles size, typically with smaller nanoparticles showing lower MIC. From their adhesion results alterations of membrane potential, inhibition of adenosine triphosphatase (ATPase) activity (resulting in inhibition of ATP synthesis) and inhibition of tRNA binding in subunit of the ribosome. This phenomenon will block instrumental metabolic processes which result in the loss of cellular integrity [40].

Due to their low reactivity, AuNPs show ion release and ROS production as a minor mechanism of action. Therefore, they need to be achieve higher concentrations to produce the same antibacterial effect as other metal-based nanoparticles (for example AgNPs) [142]. In this sense, Zhang et al. showed that to produce the small zone of inhibition for *S. aureus*, AuNPs need a concentration of 197 µg/mL, whereas AgNPs need small concentrations as 4.86 µg/mL [142].

### 5.4. Other Pharmaceutical Properties

Pharmaceutical properties of AuNPs have been recently studied. Besides their antibacterial activity, AuNPs have antioxidant and anticancer activities. AuNPs are seen as a relatively new agent in cancer therapy because these nanoparticles minimize side effects and limit the damage in healthy cells. The mechanism of action is not well known, but researches have reported cellular internalization of AuNPs by cancer cells due to their surface specific characteristics. Due to the optical and electronic properties of gold, AuNPs have been also studied for biomedical applications, such as nanodelivery (drugs, genes), imaging, (photoacoustic imaging, computed tomography), therapy (photothermal therapy and radiosensitization) and diagnostics (chemical and biological sensing) [143].

### 5.5. Toxicity Assessment

Despite the potential applicability of AuNPs, some doubts remain about their potential toxicity. The mainstream opinion that AuNPs are non-toxic is recently disputable. The potential toxicity in vitro and in vivo of AuNPs appears to be multi-faceted and difficult to predict. Some authors investigated in vitro toxicity of AuNPs, and the results showed that these nanoparticles induce generation of endogenous ROS after entering the cells and then lead to further oxidative stress-related cytotoxicity such as DNA damage, cell death and cell cycle arrest in consequence. As an example of an in vitro cell viability assay, AuNPs with a size of 15–20 nm decrease cell viability from 100% at a concentration of 0.1 ppm to less than 40% at a concentration of 10 ppm, showing their potential toxicity [144]. On the other hand, some researches have reported the non-toxicity of the AuNPs in vitro testing different cell lines, nanoparticle shape and surface groups, and doses [145].

The in vivo toxicity has been studied in some animal models as mice, rat, zebrafish, shrimp, snail, clam and pig, assessing different nanoparticle shapes, surface groups and doses. The obtained results did not report side effects or lethal toxicity. However, most studies report higher bioaccumulation in liver, and a few authors observed accumulation in other organs like lung, brain, heart and kidneys. However, neither the mechanism of toxicity nor doses have been described yet [145].

The environmental impacts of AuNPs still remain unknown. There are some studies about phytotoxicity and aquatic toxicity. AuNPs can exert phytotoxicity to aquatic environment at a concentration of gold in the form of nanoparticles of 6 × 10^−6^ M [146]. Moreover, acute toxicity of aquatic organisms, such as fish and arthropods, has been studied. The results showed that fishes are more sensitive than smaller organisms (as daphnia), arthropods can undergo molting in order to cope with the particles adhered onto their shell. For the majority of fish species exposed to ionic gold (2.44 mg/L), 50% of mortality has been reached between 12 h and 24 h [147].

## 6. Zinc Oxide Nanoparticles (ZnONPs)

Zinc is an essential mineral involved in the catalytic activity of numerous enzymes present in the organism and is widely distributed throughout the body tissue [151]. Zinc oxide (ZnO) is a multifunctional and biocompatible semiconductor material used in the preparation of many products including plastics, paints, ceramics, batteries and as an antibacterial [152]. In the pharmaceutical field it is recognized as one of the safest materials by the Food and Drug Administration (FDA) [50]. Nanosized ZnO presents great interest in the industrial sector because of its intrinsic properties as wide bandgap (3.37 eV), high-exciton binging energy (60 MeV), high electronic conductivity, nontoxicity and chemical stability [153]. ZnONPs exhibit significant optical properties that offer NPs the ability to be used as drug delivery system, as antitumor, antibacterial, antidiabetic and as theragnostic tool. 

### 6.1. Synthesis and Production Methods

Among the most widely used techniques for synthetizing of ZnONPs are widely known the solution-based routes as chemical controlled precipitation, sol-gel method, solvothermal and hydrothermal method, method using an emulsion or microemulsion environment, among others [154]. ZnONPs are formed using as precursors mainly zinc nitrate (Zn(NO_3_)_2_), zinc sulphate (ZnSO_4_) and zinc acetate (Zn(CH_3_COO)_2_). Zinc oxide is organized spatially in two main forms: hexagonal wurtzite, the most stable form and therefore the most common and cubic zinc blende. In addition to this fixed form, after agglomeration ZnONPs may present different morphologies (e.g., nanorings, nanocombs or nanocages) which is determined by the synthetic route used [151]. The color change in the solution to yellow evidences the formation of this nanoparticles [155]. The simplified reactions of its synthesis are presented below [156].

Zn^2+^ 2OH^−^    Zn(OH)_2_ (8)

Zn(OH)_2_ + 2OH^−^  Zn(OH)_4_^−2^ (9)

Zn(OH)_4_^−2^   ZnO + 2H_2_O + 2OH^−^ (10)

Chemical strategies arise in the presence of noxious chemicals absorbed on the nanoparticles surface, producing adverse effects in medical application [154]. Biological or green synthesis consists on the use of non-toxic, environmentally friendly and safe reagents as microorganisms, enzymes and plants or plant extracts as reactive in the manufacture procedure of ZnONPs [154].

### 6.2. Pharmacokinetics

According to the administration route, the bioavailability of ZnONPs differs. In the case of intravenous injection, nanoparticles are readily available in the bloodstream, while the bioavailability is much lower after oral dose. This phenomenon is explained by a limited absorption at gastrointestinal tract level, followed by a first-pass hepatic effect [157].

The ZnONPs distribution is influenced by the route of administration and the intrinsic physicochemical properties. ZnONPs have a wide distribution to organs such as the heart, spleen, liver, kidneys and lungs, accumulating specially in liver and kidneys, their main metabolic and/or excretion sites [158].

Despite being eliminated mainly by biliary clearance and faecal excretion, some smaller ZnONPs follow a minor route based on a renal excretion [157].

### 6.3. Antibacterial Properties

ZnONPs possess antimicrobial activity against Gram-positive (*S. aureus*, *S. epidermis*, *B. subtilis*, *B. cereus*, *L. monocytogenes*, *E. faecium*) and Gram-negative (*P. aeruginosa*, *E. coli*, *K. pneumoniae*, *Salmonella* sp.) bacteria [50].

Moreover, Singh et al. compared antimicrobial and antifungal potential of zinc oxide nanoparticles. Pathogenic microorganisms selected included different bacteria, *Escherichia coli* (MTCC 443), *Staphylococcus aureus* (MTCC 3160), *Bacillus subtilis* (MTCC 441) and two fungi, *Aspergillus niger* (MTCC 281), and *Candida albicans* (MTCC 227). They show a 50% efficacy when nanoparticles are used instead of particles [159]. Antimicrobial tests carried out with different bacterial strains show than the minim inhibitory concentration (MIC) values are 50–85% lower in Gram-positive than Gram-negative bacteria.

The difference in antibacterial activity of ZnONPs towards Gram-positive and Gram-negative bacteria could be clarified due to the interaction with the cell wall. In Gram-negative bacteria, the structure of lipopolysaccharide opposes the attachment of ZnO and restrains the ions passing across the outer membrane [160].

The toxicity induced by antimicrobial drugs is due to modifications in the membrane potential through the blockage of K^+^ ion channel present in the bacteria cell membrane. ZnONPs in aqueous medium are dissolved with consequent release of Zn^2+^. Zn^2+^ ions, that are attracted to the bacterial surface causing this phenomenon. These changes increased permeability of the membranes leading to destabilization. Moreover, ROS production by nanoparticles contributes to this result [157].

Due to their charge, Zn^2+^ ions can easily penetrate the bacterial cell wall and interact with different molecules—such as lipids, proteins, and nucleic acids—disrupting important metabolic pathways. In presence of acidic pH nanoparticles shows a higher antimicrobial activity due to the increase in the dissolution and release of Zn^2+^ (as described below) [157].

ZnO + 2NaOH ^basic medium^ Na_2_ZnO_2_ + H_2_O (11)

ZnO + 2NaOH ^acidic medium^ ZnCl_2_ + H_2_O (12)

ZnCl_2_ ^aqueous medium^ Zn^+2^ + 2Cl^−^ (13)

Several authors have used ZnONPs as antimicrobial agents either as ZnO alone or in combination with drugs [161]. Recently, ZnONPs have been developed using a plant extract (*Punica granatum*) and obtaining spherical and hexagonal shapes of 32.98 nm diameter with antibacterial activity against *E. coli* and *E. faecalis* [162]. Moreover, Jayabalan et al. in their last work have demonstrated that the ZnONPs synthetized using a biological method based on the use of *Pseudomona putida*, obtaining nanoparticles with a spherical shape and an average diameter of 44.5 nm. This ZnOPNs presented antimicrobial activity against *Pseudomonas otitidis*, *Pseudomonas oleovorans*, *Acinetobacter baumannii*, *Bacillus cereus*, and *Enterococcus faecalis* using microtiter plate method and disk diffusion assay (Table 5) [163]. 

### 6.4. Other Pharmaceutical Properties

Recently ZnONPs have been found to have the potential to be used in cancer therapy since they exhibit tumoral cell selectivity showing enhanced in vitro cytotoxicity by generation of ROS. Furthermore, ZnONPs induce proinflammatory markers as interferon-c, IL-12 and tumor necrosis factor-α (TNF-α) in peripheral blood mononuclear cells [164]. Several studies demonstrated that ZnONPs cause genotoxic effects including DNA damage in neuronal and human epidermal cancer cells [156].

Different studies have assessed the effect of ZnONPs in antidiabetic activity. Bala et al. have observed a reduction of blood glucose levels when administered the ZnONPs in diabetes induced mice [165]. Other authors have shown that it results in a synergic effect when combined these nanoparticles with an antidiabetic drug as Vildagliptin in diabetic-induced rats [166]. 

Due to the ZnONPs ability to absorb radiation, they are UVA and UVB reflectors approved to be used as a physical sunblock since they are completely photostable, non-allergenic, non-irritating and non-comedogenic. Zinc oxide blocks every UVA (320–400 nm) and UVB (280–320 nm) rays [167]. Therefore, ZnONPs are used in the preparation of sun creams in combination of TiO_2_, acting as a successful UV blocker [168]. Currently, large brands of dermocosmetic that manufacture sunscreens use this type of nanoparticles mainly as physical filters in sunscreens.

### 6.5. Toxicity Assessments

Regardless of the advantages and potential applicability of nanoparticles against bacteria, some doubts remain about their potential toxicity and risk to human health.

Even though metal-based nanoparticles have been shown to be more toxic to bacteria than to eukaryotic cells, their extensive distribution and accumulation over time predicts that the same mechanisms responsible for their efficacy against bacteria may also be responsible for potential adverse effects [174].

Production of ROS by nanoparticles and generated oxidative stress have been associated with inflammatory processes responsible for many disorders, such as pulmonary diseases and liver degeneration [175].

#### 6.5.1. Pulmonary Toxicity

In the lungs, metal-based nanoparticles are involved in acute and subacute inflammatory processes with release of pro-inflammatory cytokines [176]. These inflammatory processes may be reversible depending on dose and exposure duration, but in some serious cases, they have been associated with the development of pulmonary bronchitis, emphysema, and moderate fibrosis [177].

Chung et al. detected a mild to moderate inflammation with infiltration of neutrophils, eosinophils, monocytes/macrophages, and some lymphocytes in the bronchial and exudates in the alveolar space after instillation with silver nanowires in Sprague-Dawley rat lungs [178]. Gosens et al. and Jacobsen et al. also proved the occurrence of the same effects after administration of CuONPs and ZnONPs, respectively [179]. Other authors observed dose-dependent lung inflammation with alveolitis, bronchiolitis, vacuolation of the respiratory epithelium and emphysema after short-term inhalation exposure to CuONPs [179] while Jacobsen et al. noticed a very strong inflammatory response in the lungs after exposure to ZnONPs [180].

#### 6.5.2. Hepatotoxicity

In the liver, high accumulation of metal-based nanoparticles has been associated with elevation of liver biomarkers (AST, ALT, GGT), hepatic inflammation and pro-inflammatory activation of Kupffer cells in the liver. At the same time inhibition of several cytochrome P450 enzymes (CYP1A, CYP2C, CYP2D, CYP2E1, and CYP3A) have also been reported [181]. Similarly, Almansour et al. also proved that ZnONPs have potential oxidative stress in hepatic tissues resulting in Kupffer cells hyperplasia, inflammatory cells infiltration, hepatocytes apoptosis and necrosis after performing a study where he evaluated ZnONPs hepatotoxicity in male Wistar albino rats [182].

#### 6.5.3. Nephrotoxicity

It has been observed an increase in blood urea nitrogen levels and serum creatinine levels in animals treated with CuONPs and ZnONPs respectively, with evidence of tubular epithelial cell necrosis observed in the second case. These results corroborate the hypothesis of renal dysfunction potentially induced by oxidative stress [183].

Although Ibrahim et al. reported some pathological changes in the kidney after exposure to AuNPs during work, such as alterations in glomeruli, dilated tubules, oedema exudate, mild necrosis, and infiltration of inflammatory cells; these pathological changes were considered minimal and insignificant in the kidneys, regardless of the size of the AuNPs [184].

#### 6.5.4. Neurotoxicity

It has been proven that nanoparticles may cause some degree of damage to the blood–brain barrier leading to increased permeability and facilitating its penetration into the central nervous system (CNS) [185]. Similarly, AgNP and ZnONP toxicity was also associated with the accumulation of nanoparticles in the brain, responsible for some degree of neuroinflammation and neurodegeneration caused by ROS induced CNS injury [186].

### 6.6. Other Side Effects

ZnONPs intake has been also associated with anaemia resulting from toxicity against erythrocytes which resulted in hemolysis and decreased count of red blood cells. Besides oxidative stress which is seen for all metal-based nanoparticles and to which erythrocytes are quite sensitive, ZnONPs show an additional mechanism of toxicity to red blood cells. A high intake of zinc causes a deficit of copper and iron. In rodents, iron deficiency is the most frequently reported cause of anemia in the literature [187].

After oral administration, gastrointestinal side effects have also been frequently reported. The most commonly reported gastrointestinal reactions include nausea, vomiting, diarrhea, and gastrointestinal hemorrhaging. These side effects are produced by the diffusion and passage of nanoparticles into the intestinal enterocyte and epithelium reaching the systemic circulation. In addition, the strong acid media (pH~2) favors, metallic and metal oxide nanoparticles dissolution into metal ions. They penetrate cells via ion channels and biological pumps and when they exceed tolerable range, toxic effects occur [188]. It is due to an inflammatory cell response induced by an intracellular production of ROS. These high ROS levels produce damage in the DNA of the cells and may result in cell death [189]. It was found that people without underlying health problems were less likely to develop adverse reactions when compared to people with comorbidities, such as obesity or high levels of cholesterol in the blood. Due to the extended list of adverse reactions, during the administration of metal-based nanoparticles, it may be relevant to access the patient’s health profile [190].

## 7. Comparative Overview

Although the different types of metal nanoparticles here discussed have a wide range of applications in several industries, with the discovery of their antimicrobial activity, AgNPs have taken a leading role since 2016 when they were marketed the first time. Their market is expected to exceed USD 3 billion in 2024 [191].

Although AgNPs have shown to be highly reactive and to have high antibacterial activity compared to others (e.g., CuONPs, AuNPs, and ZnONPs), their accumulation in the body and potential toxicity to the various organs may limit their use. This is the main reason why other metal-based nanoparticles are being studied. According to Bondarenko et al., MIC values for bacteria for Ag, CuO, and ZnONPs are 7.1, 200, and 500 mg/L [192]. Regarding AgNPs, acute oral exposure to AgNP at doses relevant to potential human exposure is associated with predominantly faecal elimination and not with increased toxicity [193]. On the other hand, high concentrations of AuNPs administered orally or intraperitoneally in rats, induced a decrease in body weight, red blood cells, and hematocrit. Moreover, it has been also reported that AuNPs orally administered in rodents cause significant decreases in body weight, spleen index, and red blood cells [194]. Regarding ZnONPs, during prolonged oral administrations in mice, they can cause cardiac damage [195]. CuO and ZnONPs, for example, are composed of copper and zinc, two essential microelements, which are less toxic to mammalian cells because these have mechanisms of homeostasis that regulate the concentrations of these metals inside the cells. However, in fish exposed to 0.25 mg/L copper, copper sulfate, and copper oxide nanoparticles dissolved copper damaged the fish liver and kidney more severely than copper nanoparticles [196].

AuNPs were originally investigated for their applicability as biosensors, bio-imaging, and drug delivery systems and not for their antibacterial activity. However, with the discovery of unfavorable toxicity profiles of other nanoparticles AuNPs antibacterial activity was also investigated since they are relatively inert and biocompatible [126]. Despite these advantages, AuNPs have considerably lower antibacterial activity in comparison with AgNPs.

## 8. Conclusions

Metal-based nanoparticles are being extensively used in biomedical sciences and in engineering. Their market has heavily increased over the last few years and it is not expected to decrease. We have revised the main features of AgNPs, CuONPs, AuNPs, and ZnONPs, commonly being exploited for medical and pharmaceutical applications (e.g., as antibacterial, antifungal, antiviral, antiamebial, anti-cancer, anti-angiogenic, anti-inflammatory agents). Due to their well-described antimicrobial activity against Gram-positive and Gram-negative bacteria, these particles have been proposed as alternative over traditional antibiotics to overcome bacteria resistance. Nanoparticles make use of mechanisms of action that differ from the classical treatments, with the advantage of being active against bacteria that have already developed antibiotic resistance, but also by targeting multiple biomolecules which compromises the development of resistant strains. Any potential risk of toxicological events in humans when using metal nanoparticles is attributed to their physicochemical properties, dosage, and administration route, which govern their pharmacokinetics and pharmacodynamics. As these particles may have a narrow therapeutic window, an exhaustive physicochemical characterization in the early stages of pharmaceutical development is recommended. Besides, the understanding of their in vivo behavior during preclinical and clinical trials is a vital source of information for the success of pharmaceutical development, so that failures in late phases of research and development are avoided.

## Figures and Tables

**Table 1:** Examples of green synthesis of alternative metal-based nanoparticles with potential antibacterial activity, with respective minimum inhibitory concentration (MIC) and minimum bactericidal concentration (MBC) values.

Specie	Microorganism	Morphology	Synthesis	Average Size (nm)	Activity	MIB and MIC Values	References
	**AuNPs**						
*Trichoderma hamatum*	fungus	spherical, pentagonal and hexagonal	extracellular	5–30	*P. aeruginosa; Serratia sp.; B. subtilis; S.aureus*	Data not shown	[40]
*Alternanthera bettzickiana*	plant extract	spherical	extracellular	80–120	*S. typhi; P. aeruginosa; E. Aerogenes; S. aureus; B. subtilis; M. luteus*	MIC values (expressed in µL of AuNPs): 10 µL *B. subtilis* 20 µL *S. aureus* 30 µL *M. luteus* 40 µL *E. aerogenes, S. typhi and P. aeruginosa*	[41]
*Deinococcus radiodurans*	bacteria	spherical, triangular and irregular	intra- and extracellular	~43.75	*E. coli; S. aureus*	Data not shown	[42]
*Pseudomonas veronii* AS41G	bacteria	irregular	extracellular	5–25	*E. coli; S. aureus (+)*	Data not shown	[43]
*Bacillus licheniformis*	bacteria	spherical	extracellular	20–75 (~38)	*E. coli; P. aeroginosa; B. subtilis*	Values not shown	[44]
*Fusarium oxysporum f. sp. cubense JT1*	fungus	n.a.0F	extracellular	~22	*Pseudomonas sp.*	*Data not shown*	[45]
*Stoechospermum marginatum*	algae	spherical to irregular	extracellular	18.7–93.7	*P. aeruginosa; V. cholerae; V. parahaemoluticus; S. paratyphi; P. vulgaris; S. typhi; K. pneumoniae; K. oxytoca; E. faecalis(+);*	AuNPs more effective against *E. faecalis* > K. pneumoniae. Non-effective against *E. coli*	[46]
*Streptomyces viridogens (HM10)*	bacteria	spherical and rod	intracellular	18–20	*E. coli; S. aureus*	Data not shown	[47]
	**CuNPs**						
*Shewanella loihica PV-4*	bacteria	spherical	extracellular	10–16	*E. coli*	100 µg/mL Cu-NPs inhibits 86% of the bacteria	[48]
	**SeNPs**						
*Enterococcus faecalis*	bacteria	spherical	extracellular	29–195 (~99)	*S. aureus* (no observed activity against *P. aeruginosa, B. subtilis and E. coli*)	Data not shown	[49]
	**ZnONPs**						
*Glycosmis pentaphylla*	plant extract	spherical	extracellular	32–36	*S. dysenteriae; S. paratyphi;* *S. aureus; B. cereus*	At 100 µg/mL maximum inhibition is observed	[50]
*Suaeda aegyptiaca*	plant extract	spherical	extracellular	~60	*P. aeruginosa; E. coli;* *S. aureus; B. subtilis*	*P. aeruginosa* MIC and MBC: 0.19–0.78 mg/mL *E. coli* MIC: 1.56–12.50 mg/mL MBC: 6.25–12.50 mg/mL *S. aureus* MIC and MBC: 0.39–1.56 mg/mL *B. subtilis* MIC: 0.19–0.39 mg/mL MBC: 0.78–12.50 mg/mL	[51]
*Pichia kudriavzevii*	fungus	hexagonal	extracellular	10–61	*E. coli(+); S. marcescens;* *B. subtilis(+); S. aureus (+); S. epidermis (++)*	Data not shown	[52]
*Jacaranda mimosifolia*	plant extract	spherical	extracellular	2–4	*E. coli;* *E. faecium*	Data not shown	[53]
	**CuONPs**						
*Cystoseira trinodis*	algae	spherical	intracellular	6–7.8	*E. coli; S. typhi; E. faecalis; S. aureus; B. subtilis; S. faecalis*	*E. coli* and *S. aureus* MIC: 2.5 μg/mL *E. faecalis* MIC: 5 μg/mL *S. typhimurium* MIC: 10 μg/mL	[54]

^2^ n.a. – information not available.

**Table 2:** Antibacterial applications of silver-based nanoparticles, with respective mminimum inhibitory concentration (MIC) and minimum bactericidal concentration (MBC) values.

Nanoparticles Efficacy	Physicochemical Characteristics of the Nanoparticles	Production Method	Therapeutic Efficacy	MIB and MIC Values	Reference
Coliforms bacteria in water and fecal media	Monodispersed spherical AgNPs Average size 20–60 nm ζ-potential (−30 to −15) mV	*(Chemical reduction)* Green method from extracts of *Olea Europaea* leaves (Leccino and Carolea), pH 7 or 8	Antibacterial activity evaluated with total bacteria detection by plate count techniques. Conducted trials of toxicology and cytotoxicity (WST-8 assay, lactate dehydrogenase (LDH) assay, comet assay)	Data not shown	[105]
Human pathogenic Gram-positive and Gram-negative bacteria: *Staphylococcus aureus, Escherichia coli, Klebsiella pneumoniae, Pseudomonas aeruginosa* and methicillin-resistant *Staphylococcus aureus (MRSA))*	Spherical or rarely polygonal AgNPs Average size 44 nm	*(Chemical reduction)* Green method AgNPs were synthesized using *Picea abies L*. stem bark extract, and sing different surfactants	Effective antioxidant activity	*Staphylococcus* *Aureus:* (MIC 0.05 mg/mL, MBC 1.57 mg/mL) *MRSA*: MIC 0.09 mg/mL, MCB 0.25 mg/mL) *E. coli* MIC: 0.23 mg/mL, MCB 0.31 mg/mL *Klebsiella pneumoniae* MIC 0.63 mg/mL, MCB: 1.18 mg/mL *Pseudomonas aeruginosa* MIC 0.16 mg/mL, MCB 0.31 mg/mL	[106]
*Staphylococcus aureus, Escherichia coli*, and *Pseudomonas aeruginosa*	Spherical shape Average size 430 nm ζ-potential −15.2 mV	*(Chemical reduction)* Green method. synthesized using terpenes rich extract of *Lantana camara L.* leaves	Antibacterial aactivity assessed using agar-well diffusion method Conducted trials of Brine shrimp cytotoxicity and antioxidant potential	Data not shown	[107]
*Staphylococcus aureus, Escherichia coli*	Spherical shape Average size between 10–26 nm	*(Chemical reduction)* Green method. AgNPs were synthesized using *Acalypha wilkesiana* extract	Agar-well diffusion method was used to evaluate antibacterial activity	Data not shown	[108]
*Staphylococcus aureus*, *Escherichia coli* (Extended-Spectrum Beta-lactamase *(ESBL*), and MRSA	Average size 77.68 ± 33.95 nm ζ- potential −34.6 ± 12.7 mV UV–Vis wavelength: 420 nm	*(Fungus-mediated Synthesis)* Green method. AgNPs were synthesized using *Fusarium oxysporum*	MIC, antibacterial combination assay Antimicrobial disk susceptibility test and time-kill curve assay used to evaluate antibacterial activity. Also conducted trials of cytotoxicity assay in human red blood cells	*MRSA* MIC 0.212 mg/mL *ESBL* MIC 0.106 mg/mL	[109]
*Escherichia coli, Salmonella typhi, Staphylococcus aureus,* *Vibrio cholerae, Enterococcus faecalis,* *Hafnia alvei, Acinetobacter baumannii*	Average size: first method: 428.2 ± 197.0 second method: 190.1 ± 102nm Polydispersity index: 0.4 ζ-Potential first method −22.1 ± 0.9 and second method −26.1 ± 1.4 mV, UV–Vis wavelength 412 and 418 nm.	*(Chemical reduction)* Green method. AgNPs were synthesized using *Andrographis paniculate*, aqueous, and ethanolic extracts	The zone of inhibition (ZOI), MIC, trypan blue dye exclusion assay, also conducted trials of CellToxTm green assay, LPO assay, hemocompatibility assay and in vivo intravenous delivery of AgNPs and Investigation of liver and kidney function biomarkers	*S. typhi* MIC 0.125 and 0.250 μg/mL *H. alvei* MIC 0.125 and 0.125 μg/mL *E. faecalis* MIC 0.250 and 0.250 μg/mL *A. baumannii* MIC 0.250 and 0.125 μg/mL *E. coli* MIC 0.125 and 0.250 μg/mL *V. cholera* MIC 0.125 and 0.125 μg/mL	[110]
*Staphylococcus aureus,* *Bacillus subtilis*, and *Escherichia coli*	Spherical shape Average size 13.2 ± 2.9 nm ζ-potential −16.6 mV UV–Vis wavelength 420 nm	*(bacterial-mediated* *Synthesis)* Green method. AgNPs were synthesized using *acidophilic actinobacterial SH11*	Disc diffusion, MIC and LIVE/DEAD analyses to evaluate antibacterial activity	*S. aureus* MIC 40 μg/mL *E. coli* MIC 70 μg/mL *B. subtilis* MIC 40 μg/ml	[111]
*Staphylococcus aureus,* MRSA, *Escherichia coli*, and *Pseudomonas aeruginosa*	Average size between 6.28–9.84 nm, UV–Vis wavelength range of 391– 403 nm	*(Chemical reduction)* Method into the lamellar space layer of montmorillonite/chitosan (MMT/Cts) on using NaBH4	Disc diffusion method to evaluate antibacterial activity	Data not shown	[112]
*Bacillus subtilis* and MRSA	Average size between 10 and 35 nm Polydispersity index 0.2, ζ-potential of −30 mV UV–Vis wavelength of 421 nm	*(bacterial-mediated Synthesis)* synthesized AgNps from the exopolysaccharide of recently recovered bacterial strain CEES51	Zone Inhibition Assay, MIC, MBC, Antibiofilm activity determination, colony-forming unit determination to estimate the bacterial susceptibility against AgNPs, intracellular reactive oxygen species production by AgNPs inside bacterial cells	*B. subtilis* MIC 10 μg/mL, MBC 50 μg/mL *MRSA* MIC 10 μg/mL, MBC 12.5 μg/ml	[113]
*Vibrio natriegens*	Average size 10 ± 5 nm, 30 ± 5 nm, 60 ± 5 nm, 90 ± 5 nm UV–Vis wavelength ranged from 400–420 nm	*(Chemical reduction)* Green method. AgNPs of different size were synthesized using *casein hydroly- sate* as a reducing reagent and sodium hydroxide (NaOH) as a catalyst	MIC, MCB, reactive oxygen species production by AgNps inside bacterial cells	MIC 1.0–11.5 μg/mL MBC 1.1–11.7 μg/ml	[114]
*Staphylococcus aureus* and *Escherichia coli*	Average size 20 nm UV–Vis wavelength of 390 nm	*(Chemical reduction)* Green method AgNPs were synthesized using Ultrasound assisted fabrication and fenugreek seed extract as a reducing and capping agent	The agar diffusion method was used for the antimicrobial assay. And the antioxidant activity	Data not shown	[115]
*Staphylococcus aureus, Shigella dysenteriae*, and *Salmonella typhi*	Average size from 60 to 80 nm	*(fungus-mediated* *Synthesis)* Green method. AgNPs were synthesized using *Penicillium oxalicum*	Antimicrobial potential in liquid broth by optical density measurements, and disc diffusion method	Data not shown	[116]
*Pseudomonas aeruginosa, Klebsiella pneumoniae*, and *Escherichia coli* MRSA	Average size 10 to 40 nm ζ-potential −29 ± 0.11 mV	*(Chemical reduction)* Green method. AgNPs were synthesized using lyophilized *Seabuckthorn*	MIC, MCB, evaluation of *P. aeruginosa* biofilm, anti-quorum sensing inhibition assay. Also conducted trials of cytotoxicity assay with human dermal fibroblast	*P. aeruginosa* MIC 2 μg/mL, MBC 4 μg/mL *E. coli* MIC 4 μg/mL, MBC 8 μg/mL *S. aureus* MIC 4 μg/mL, MBC 8 μg/mL *K. pneumoniae* MIC 8 μg/mL, MBC 16 μg/mL	[117]
*Escherichia coli*- 25922 and multidrug-resistant pathogens of *Pseudomonas aeruginosa* and *Acinetobacter baumannii*	Spherical shape Average size from 35 to 50 nm, UV–Vis wavelength of 326 nm	*(Chemical reduction)* Green method. AgNPs were synthesized using *Sisymbrium irio* extract	The agar diffusion method was used for the antimicrobial assay	Data not shown	[118]
*Bacillus cereus,* *Staphylococcus aureus, Micrococcus Luteus,* *Bacillus Subtilis, Enerococcus Sp.* *Pseudomonas aeruginosa, Salmonella typhi, Escherichia coli,* and *Klebsiella pneumonia*	Spherical shape Average size 10 nm UV–Vis wavelength of 432 nm	*(Chemical reduction)* Green method. AgNPs were synthesized using *Tamarindus indica* natural fruit extract	The agar diffusion method was used for the antimicrobial assay	Data not shown	[119]
*Escherichia coli, Bacillus subtilis, Pseudomonous fluorescence* and *Salmonella typhi*	Average size 21 nm ζ-potential −32 mV UV–Vis wavelength of 421nm	*(Chemical reduction)* Green method. AgNPs were synthesized using *Ficus religiosa* leaf extract	Kirby–Bauer Disk diffusion method and the growth inhibition curve of *E. coli* was examined after the exposure of AgNPs. Also conducted trials of anti-cancer activity and in vivo toxicity	Data not shown	[120]

**Table 3:** Antibacterial applications of Cu and CuO-based nanoparticles, with respective minimum inhibitory concentration (MIC) and minimum bactericidal concentration (MBC) values.

Nanoparticles Efficacy	Physicochemical Characteristics of the Nanoparticles	Production Method	Therapeutic Efficacy	MIB and MIC Values	Reference
*Staphylococcus aureus Pseudomonas aeruginosa*	Data not shown	Data not shown	Ultrasound increased the antibacterial effect of CuO nanoparticles against *S. aureus* and *P. aeruginosa*	Data not shown	[129]
*E. coli* *S. epiderdimis* *methicillin 655 resistant S.s aureus (superbug MRSA) isolate* *Spore-forming Bacillus megatarium*	Nanoparticles ranged from 30 to 60 nm	Data not shown	Reaction of copper nanoparticles of 100 nm with B. subtilis showed the highest susceptibility (Z = 0.0734 mL/μg) whereas the reaction of silver nanoparticles of 40 nm with E. coli showed the lowest one (Z = 0.0236 mL/μg)	Data not shown	[130]
*B. megatarium,* *S. epidermidis,* *E. coli* *MRSA*	Average size of 1.36 ± 0.6 nm	CuCl_2_ as the precursor, D (+) glucose as the reducing agent, soluble starch as the NP stabilizing agent	Cu1X and Cu10X kill *B. megatarium, S. epidermidis, E. coli and MRSA*	Data not shown	[131]
*E. coli* *S. aureus*	Spherical morphology and a narrow size distribution with 7 and 14 nm	Mechanochemical method using two different Cu-containing precursors (i.e., CuSO4·5H2O and CuCl2·2H2O)	CuCl_2_·2H_2_O derived nanoparticles showed more antibacterial activity than CuSO_4_.5H_2_O derived nanoparticles	*E. coli* MIC:3.75 mg/mL *S. aureus* MIC: 2.50 mg/mL	[132]

**Table 4:** Antibacterial applications of Au-based nanoparticles, with respective minimum inhibitory concentration (MIC) and minimum bactericidal concentration (MBC) values.

Nanoparticles Efficacy	Physicochemical Characteristics of the Nanoparticles	Production Method	Therapeutic Efficacy (Tests Employed)	MIB and MIC Values	Reference
*P. aeruginosa*	Average size 18.32 nm	Biological method (extract of *A. comosus)*	Disc diffusion method	MIC, MIB: 4 μg/mL	[148]
*S. aureus*				MIC: 3.92 μg/mL	
*E. coli*	Average size 150 nm	Biological method (extract of *M. piperita*)	Disc diffusion method	MIB: 12–16 μg/mL MIC: 4 μg/mL	[149]
*K. pneumoniae*	Average size 77.13 and 38.86 (due to extraction method)	Biological method (extract of *G. elongate*)	Standard agar well diffusion method	MIC: 3.3 μg/mL	[148]
*S. typhimurium*	Average size 25 to 35 nm	Biological method (extract of *S. brachiate)*	Disc diffusion method	MIC, MIB: 8 μg/mL	[150]
*K. oxytoca*	Average size 18.7 to 93.7 nm	Biological method (extract of *S. marginatum*)	Agar well diffusion method	Data not shown	[46]
*E. faecalis*				Data not shown	
*V. cholerae*				Data not shown	
*S. paratyphii*				Data not shown	
*V. parahaemolyticus*				Data not shown	
*P. vulgaris*				Data not shown	
*B. subtilis*	Average size 6 to 40 nm	Chemical method [sodium borohydride (NaBH4) as a reducing agent+	Enzyme-linked immunosorbent assay (ELISA)	MIC 7.56 μg/mL	[148]

**Table 5:** Antibacterial applications of zinc oxide nanoparticles, with respective mminimum inhibitory concentration (MIC) and minimum bactericidal concentration (MBC) values.

Organism and Specie against the Nanoparticles are Effective	Physicochemical Characteristics of the Nanoparticles	Production Method	Therapeutic Efficacy Assessment	MIB and MIC Values	Reference
*Escherichia coli* *Enterococcus faecalis*	Spherical and hexagonal-shaped UV–Vis absorption 32.98 nm (600 °C) UV–Vis absorption 81.84 nm (700 °C)	Green method Biosynthesis of ZnO-NPs using *Punica granatum* fruit peels extract	Antimicrobial susceptibility test shows effective antibacterial activities against two strains of bacteria Cell proliferation assay shows selective toxicity towards colon cancer cells (HCT116) and proved non-toxic to normal cell (CCD112)	MIC *E. coli* – 64.53 µg/mL MIC *E. faecalis* – 22.09 µg/mL	[162]
*Pseudomonas otitidis* *Pseudomonas oleovorans Acinetobacter baumannii* *Bacillus cereus Enterococcus faecalis*	Spherical shape Average size 25–45 nm	Green method Biogenic synthesis of ZnO NPs using *Pseudomonas putida* broth culture	Antibacterial microsomal triglyceride transfer protein assay shows effective antibacterial activities against all strains of bacteria	MIC 10 µg/mL in all bacteria	[163]
*Staphylococcus aureus*	Hexagonal shape UV–Vis absorption 25.57 nm ζ-potential −20.9 mV	Green method Biosynthesis of ZnO-NPs using *Cinnamomum* *Tamala* leaf extract	Broth dilution assay, protein leakage analysis, membrane stability analysis, and growth curve analysis show a time and concentration dependent reduction in bacterial growth	MIC 40 µg/mL	[169]
*Escherichia coli* *Listeria monocytogenes*	Uniform rod-shape Average size 20-30 nm diameter, 100–150 nm length	Green method Synthesis using KOH as a hydrolysing agent	The viable colony count method shows effective antibacterial activities against both strains of bacteria	Data not shown	[170]
*Escherichia coli*	Spherical shape Average size 60–80 nm	Green method Phyto-assisted synthesis of ZnO-NPs using *Cassia* *alata* fresh leaves	Growth kinetic assay demonstrated bacteriostatic effect	MIC 20 µg/mL	[157,171]
*Bacillus cereus* *Bacillus subtilis* *Escherichia coli* *Klebsiella pneumoniae* *Staphylococcus aureus* *Serratia marcescens*	Needle like shape Average size 90–110 nm	Green method Phyto-assisted synthesis of ZnO-NPs using *Berberis aristata* leaf extract	Antibacterial activity assay shows effective antibacterial activities against all strains of bacteria and MIC was determinate. The maximum activity was found against *Bacillus subtilis*	MIC *B. cereus –* 128 µg/mL *B. subtilis –* 64 µg/mL *E. coli -* 256 µg/mL *K. pneumoniae* – 256 µg/mL *S. aureus -* 128 µg/mL *S. marcescens* 64 µg/mL	[172]
*Staphylococcus aureus* *Escherichia coli* *Salmonella paratyphi*	Spherical shape Average size 20–50 nm	Green method Biosynthesis of ZnO-NPs using aqueous *Tabermaemontana divaricata* leaf extract	Antibacterial activity assay shows effective antibacterial activities against all strains of bacteria	Data not shown	[173]
